# Optical Coherence Tomography Observation of Gonio Structures during Microhook Ab Interno Trabeculotomy

**DOI:** 10.1155/2017/6310835

**Published:** 2017-08-06

**Authors:** Masaki Tanito

**Affiliations:** Division of Ophthalmology, Matsue Red Cross Hospital, Matsue, Japan

## Abstract

**Introduction:**

Intraoperative observation of ocular structures using microscope-integrated optical coherence tomography (iOCT) has been adopted recently. I report my initial feasibility assessment of iOCT for the incised trabecular meshwork observation during microhook ab interno trabeculotomy.

**Case Series:**

Both the nasal and temporal sides or either side of the trabecular meshwork/inner wall of Schlemm's canal was incised more than 3 clock hours. After then, under observation using a Swan-Jacob gonioprism lens with the real-time 5-line scan mode, OCT images of the area were successfully acquired in 10 (83%) of 12 sides in nine eyes. Based on the appearance of the acquired images of the 10 sides, the trabeculotomy cleft could be classified into three incisional patterns, that is, six (60%) anterior-opening patterns (posterior-based flap), three (30%) middle-opening patterns (posterior- and anterior-based flaps), and one (10%) posterior-opening pattern (anterior-based flap), according to the predominant locations of the trabecular meshwork flaps.

**Conclusion:**

Intraoperative observation of the gonio structures including the trabeculotomy cleft was feasible using the RESCAN 700 in combination with a gonioprism.

## 1. Introduction

Recently, optical coherence tomography (OCT) was integrated into surgical microscopes and is no longer handheld. So far, intraoperative observation of ocular microarchitectural structures using OCT (iOCT) has been adopted in the assessment of macular hole and epiretinal membrane during vitreoretinal surgery, visualization of the donor cornea during endothelial keratoplasty, and evaluation of the intraocular lens position during cataract surgery [1–4]; however, its feasibility was not fully assessed in glaucoma surgery.

I and collaborators initially reported the case of both eyes of one patient with steroid-induced glaucoma who underwent a novel ab interno trabeculotomy, which we referred to as microhook ab interno trabeculotomy (*μ*LOT) [5]. Because of the substantial IOP decrease in that case and less ocular surface invasiveness, we began to perform the procedure in other cases and reported the early postoperative results and safety profile of *μ*LOT in an initial case series [6]. In that case series, at the final 6-month evaluation, *μ*LOT alone decreased the IOP from the preoperative value of 25.9 mmHg to 14.7 mmHg, a 43% decrease.

I report my initial feasibility assessment of iOCT for the incised trabecular meshwork observation during microhook ab interno trabeculotomy (*μ*LOT), a novel minimally invasive glaucoma surgery [5, 6].

## 2. Case Series

This observational case series included consecutively 9 glaucoma eyes from 9 subjects (mean ± standard deviation age of 68.3 ± 8.2 years; 6 males and 3 females) who received *μ*LOT for the reduction of IOP in July 2016. The study adhered to the tenets of the Declaration of Helsinki; the institutional review board of Matsue Red Cross Hospital reviewed and approved the research. Preoperatively, all subjects provided written informed consent for surgery and use of clinical data regarding the glaucoma treatment obtained during the follow-up periods. The demographic data of subjects were glaucoma types of 5 (56%) primary open-angle glaucoma, 1 (11%) pseudoexfoliation glaucoma, 1 (11%) steroid-induced glaucoma, 1 (11%) uveitic glaucoma, and 1 (11%) mixed mechanism glaucoma; angle-opening grade of 3.2 ± 0.7 in Shaffer grading; lens status of 8 (89%) phakic and 1 (11%) pseudophakic eyes; and 1 eye that had a history of previous glaucoma surgery (ab externo trabeculotomy). The procedure included *μ*LOT alone in 33 (33%) eyes and *μ*LOT combined with cataract surgery in 6 (67%) eyes.

**Figure 1:**
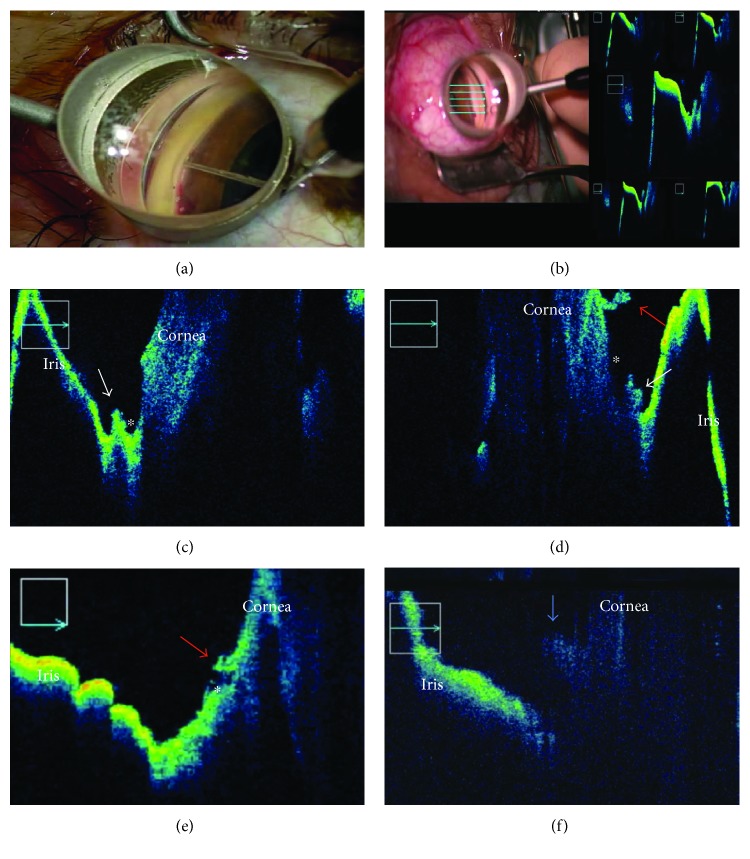
iOCT during *μ*LOT. (a) Intraoperative findings during microhook ab interno trabeculotomy. Under observation using a Swan-Jacob gonioprism lens, a microhook is inserted into Schlemm's canal. In this case of the left eye, temporal angle is being incised with the microhook that was inserted from the nasal corneal port. (b) Intraoperative observation of the incised trabecular meshwork and inner wall of Schlemm's canal by 5-line scans of the RESCAN 700 in combination with a Swan-Jacob gonioprism lens. In this case of the right eye, temporal angle is visualized with iOCT. (c, d, e) Based on the flap locations (white and red arrows), the trabeculotomy cleft is classified into an anterior incisional pattern (c) (seen with posterior-based flaps predominantly), middle incisional pattern (d) (seen with posterior- and anterior-based flaps), or posterior incisional pattern (e) (seen with anterior-based flap predominantly). (f) The trabeculotomy cleft is unclear because the OCT signal is blocked by a blood clot. The white arrow, a posterior-based flap; the red arrow, an anterior-based flap; the blue arrow, a blood clot; and the asterisk, the lumen of Schlemm's canal.

The *μ*LOT was performed through two corneal side ports as reported previously [6]. Briefly, a spatula-shaped microhook designed specifically for use during *μ*LOT was used (M-2215, Inami, Tokyo, Japan). Viscoelastic material (1% sodium hyaluronate, Opegan Hi, Santen Pharmaceutical) was injected into the anterior chamber through the clear corneal ports created using a 20-gauge microvitreoretinal knife (Mani, Utsunomiya, Japan) at the 2 to 3 and 9 to 10 o'clock positions. A microhook was inserted into the anterior chamber through the corneal port using a Swan-Jacob gonioprism lens (Ocular Instruments, Bellevue, WA) to observe the angle opposite to the corneal port. The tip of the microhook then was inserted into Schlemm's canal and moved circumferentially to incise the inner wall of Schlemm's canal and trabecular meshwork over 3 clock hours (Figure 1(a)). Using the same procedure, LOT was performed in the opposite angle using a microhook inserted through the other corneal port.

iOCT then was performed to assess the gonio structures using spectral-domain iOCT (RESCAN 700, Carl Zeiss Meditec Japan, Tokyo, Japan); however, the angle structure was not visualized through the limbal tissue because of poor penetration of the light source. Alternatively, under observation using a Swan-Jacob gonioprism lens with the real-time 5-line scan mode, I successfully acquired OCT images of the area I intended to observe in 10 (83%) of 12 sides (i.e., nasal and/or temporal angles) in nine eyes in which iOCT was performed (Figure 1(b), Supplementary Video 1 available online at https://doi.org/10.1155/2017/6310835). After the iOCT imaging, the viscoelastic material was aspirated bimanually and the corneal ports were closed by corneal stromal hydration.

At the final follow-up of 7.0 ± 1.7 months postsurgically, the baseline IOP of 20.6 ± 7.9 mmHg reduced to 14.9 ± 2.6 mmHg (25% reduction; *p* = 0.0078, Wilcoxon signed-rank test) while the baseline number of glaucoma medication of 3.3 ± 0.9 unchanged to 2.9 ± 1.0 (*p* = 0.2500). Baseline best-corrected visual acuity of 0.2 ± 0.6 in the logarithm of the minimum angle of resolution improved to 0.0 ± 0.1 (*p* = 0.0313) with no eye decreased visual acuity. Other than perisurgical hyphema, no surgery-related complication was recorded; one eye (uveitic glaucoma) required Ahmed glaucoma valve implantation at 5 months after *μ*LOT because of insufficient IOP reduction.

Based on the appearance of the acquired images of the 10 sides, the trabeculotomy cleft could be classified into three incisional patterns, that is, six (60%) anterior-opening patterns (Figure 1(c), posterior-based flap), three (30%) middle-opening patterns (Figure 1(d), posterior- and anterior-based flaps), and one (10%) posterior-opening pattern (Figure 1(e), anterior-based flap), according to the predominant locations of the trabecular meshwork flaps.

## 3. Discussion

Although previous reports have suggested that good visualization of the gonio structures can be achieved through a deep sclerectomy window during canaloplasty [7], my current experience indicated that Schlemm's canal cannot be visualized through the full-thickness sclera because of poor penetration of the RESCAN 700 light source. I observed a trabeculotomy cleft and the lumen of Schlemm's canal using both the RESCAN 700 and a gonioprism. In that region, where refluxed blood from the aqueous vein was presented, the trabeculotomy cleft was sometimes unclear due to blockage of the OCT signal (Figure 1(f), blue arrow). Even in such region, the 5-line scan allowed visualization of the cleft at least in part in all 10 sides when the OCT images of the region of interest were obtained. Because the iOCT itself is the noncontact method and the gonioprism use is required to perform *μ*LOT, application of iOCT in combination with a gonioprism during *μ*LOT seems a safe procedure. In two (17%) sides, no OCT image was obtained due to the lengthy time required to frame/focus the image. Previously, successful iOCT images were obtained in 224 (99%) of 227 eyes, most during corneal or vitreoretinal surgeries [1]. Thus, iOCT of the gonio structures performed in combination with a gonioprism requires some experience for successful image acquisition.

**Figure 2:**
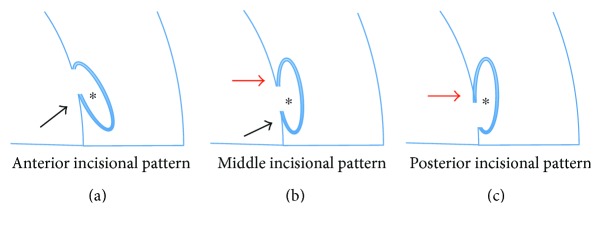
Schematic drawings of three patterns of trabeculotomy cleft. (a, b, c) Based on the flap locations (black and red arrows), the trabeculotomy cleft is classified into an anterior (a), middle (b), or posterior (c) incisional patterns. The black and red arrows indicate posterior- and anterior-based flaps, respectively. The asterisk indicates the lumen of Schlemm's canal.

I morphologically classified the appearance of the trabeculotomy cleft into three groups (Figure 2), and the cleft at the anterior edge of the trabecular meshwork was the most frequent pattern after the incision using a microhook; this should associate with the significant IOP reduction after *μ*LOT [5, 6], although the association between the patterns and surgical efficacy has not been clarified yet. Recently, based on postoperative observation using anterior segment OCT, a possible connection between the anterior chamber and suprachoroidal space through the iris root was reported in cases after trabectome surgery [8]. Although I did not find evidence of cyclodialysis or dissociation of the pectinate ligaments of the iridocorneal angle in our cases, iOCT can be used to elucidate the mechanism of the phenomenon reported by Akagi et al.

## 4. Conclusion

Intraoperative observation of the gonio structures including the trabeculotomy cleft was feasible using the RESCAN 700 in combination with a gonioprism. The reported technique might be useful to confirm the proper opening of the inner wall of Schlemm's canal during trabeculotomy.

## Supplementary Material

Video 1. Real-time observation of the incised trabecular meshwork using RESCAN 700 OCT in combination with a gonioprism.

## Additional Points

*Authorship*. The named author meets the International Committee of Medical Journal Editors (ICMJE) criteria for authorship of this manuscript, takes responsibility for the integrity of the work as a whole, and has given final approval to the version to be published.
